# No guts, no glory: underestimating the benefits of providing children with mechanistic details

**DOI:** 10.1038/s41539-021-00108-5

**Published:** 2021-10-22

**Authors:** Aaron Chuey, Amanda McCarthy, Kristi Lockhart, Emmanuel Trouche, Mark Sheskin, Frank Keil

**Affiliations:** 1 Stanford University Psychology Department, 450 Jane Stanford Way, Stanford, CA 94305 USA; 2 Yale University Psychology Department, 2 Hillhouse Ave, New Haven, CT 06520 USA; 3 Mohammed VI Polytechnic University, Lot 660, Hay Moulay Rachid, 43150 Benguerir, Morocco; 4 Minerva University, College of Social Science, 1145 Market St, San Francisco, CA 94103 USA

**Keywords:** Human behaviour, Human behaviour

## Abstract

Previous research shows that children effectively extract and utilize causal information, yet we find that adults doubt children’s ability to understand complex mechanisms. Since adults themselves struggle to explain how everyday objects work, why expect more from children? Although remembering details may prove difficult, we argue that exposure to mechanism benefits children via the formation of abstract causal knowledge that supports epistemic evaluation. We tested 240 6–9 year-olds’ memory for concrete details and the ability to distinguish expertise before, immediately after, or a week after viewing a video about how combustion engines work. By around age 8, children who saw the video remembered mechanistic details and were better able to detect car-engine experts. Beyond detailed knowledge, the current results suggest that children also acquired an abstracted sense of how systems work that can facilitate epistemic reasoning.

## Introduction

College-level science and engineering courses routinely simplify causal mechanisms, employing idealizations like frictionless surfaces, ideal gases, and perfectly inelastic interactions. Educators often extend these simplifying practices to children by eliminating mechanistic details altogether in favor of isolated facts, high-level functions, methodology, and the nature of science^1–3^. After all, mechanistic details seem far beyond children’s grasp, given that most adults cannot recognize, let alone provide, simplified explanations for how everyday objects work, despite their confidence otherwise^4,5^. For example, regular adult bicycle users in the United Kingdom appeared to know surprisingly little about how bicycles actually work, often endorsing drawings where the chain went around both the front and rear wheels^6^. Yet, omitting discussions of the “guts” that explain how things work contradicts children’s ability to reason abstractly about causal systems as well as their early information-seeking behaviors^7^.

Even infants selectively explore in order to learn about the causal structure of their environment^8,9^. More broadly, young children selectively attend to patterns of cause and effect^10^ as well as properties that afford useful interventions on systems^11,12^. Children’s information-seeking preferences also guide their usage of language. Children as young as three repeatedly request details about cause and effect through “why” and “how” questions^13–16^, behaviors that appear to be driven in part by a desire for mechanistic information^17^. Likewise, children’s own explanations tend to focus on causal relationships and can improve their memory for causal information^18–20^. Together, these findings suggest that from an early age, children actively seek causal information and are sensitive to the causal properties and affordances of objects.

Children’s interest in causal information not only enriches their own knowledge, it also influences how they reason about knowledge in other minds. Children as young as six view those possessing mechanistic knowledge as having broader, deeper, and more generalizable knowledge^21,22^. While the representations underlying such epistemic inferences are currently unclear, the way that children generalize mechanistic knowledge selectively within, but not across, domains (e.g., knowing how a clock works implies knowledge of machines but not of flowers) suggests they are able to represent causal properties shared among related kinds.

Children themselves reason mechanistically by the early elementary school years^23^. Exposure to mechanisms could contribute to the formation of such representations, even if the mechanisms themselves are forgotten. For example, young children acquire a “meta-knowledge” of the relative causal complexity of devices and biological systems, despite near total ignorance of how these systems actually work^24^. Beyond complexity, children also appear to acquire other kinds of intuitions about the broad causal patterns implicit in mechanistic descriptions. Here we ask whether children acquire three casual intuitions about internal combustion engines via exposure to mechanistic details: the transient but necessary containment of fluid, the synchronized operation of parts, and the decentralized control of parts. In addition to abstracting away from concrete details, these kinds of mechanistic representations often persist in memory longer^25–27^. Ironically, presenting richly detailed causal mechanisms might promote learning of higher-level causal patterns that endure in memory, while the very details from which they were abstracted decay.

Recent work has begun to examine the impact of causally focused instruction on children’s abstract knowledge. In one study, a group of eight- and nine-year-old students were taught about a complex topic, atomic–molecular theory, over the course of ten weeks^28^. Instead of focusing merely on what atoms and molecules are, an instructor taught children how and why certain materials, and by extension the atoms and molecules that compose them, possess certain properties. At the end of the weekly sessions, children’s abstract understanding of atomic theory and their curiosity for scientific topics drastically improved. Thus, following a relatively short period of causally focused instruction on atomic theory, children as young as eight were able to explain patterns in the periodic table in terms of electron-shell structures and could predict bonding sequences between novel configurations of atoms and molecules. Even more strikingly, children’s knowledge persisted for at least a year with no additional instruction.

In another set of studies^29^, four- and five-year-olds attempted to turn on a fan by manipulating a simple circuit, while a parent or experimenter provided mechanistic or nonmechanistic answers to their questions. Children who received mechanistic information were not only more likely to activate the fan, but also to succeed on a second task involving a novel circuit. These results suggest that informal exposure to mechanistic information aids children’s ability to reason abstractly about how a circuit system works and to generalize that knowledge to similar systems.

Although these studies demonstrate the impact of mechanistic information on children’s abstract knowledge and reasoning ability, they involved extended, child-centered dialog that varied with each child. In real-world contexts, young children are often passive viewers of mechanistic information, unable to ask sequences of how-and-why questions or receive feedback from others. Moreover, previous work has not attributed a particular set of causal abstractions to children nor systematically measured them. Can specific causal abstractions be measured in tightly controlled experimental settings without tailored interactions between teacher and student, thereby ensuring information is shared with all participants uniformly? Further, can children even form such abstractions from only brief exposure to mechanistic information?

We addressed these questions by showing children a short video designed to teach college-level adults about a complex device—an internal combustion engine. The video shows more than one hundred discrete parts, and, over the course of seven minutes, thousands of distinct causal relations between those parts. Afterward, we measured children’s memory for concrete information explicitly mentioned in the video as well as their understanding of abstract causal patterns implicit (but not explicitly stated) in the video. We assessed children’s causal abstractions by asking children to select which of two individuals was a “real” car engine expert. Crucially, one individual provided a statement that aligned with an abstract aspect of how a car engine works (e.g., when one part goes faster, all the other parts have to go faster too), while the other espoused a plausible alternative (e.g., when some parts go faster, other parts have to slow down). Therefore, children’s success on this task depends on whether they represent a particular abstract causal property of car engines. We compared performance between children who did not view the video, those who viewed the video and were immediately tested, and those who viewed the video but were tested a week later.

The ability to infer abstract patterns from rich mechanistic information and apply this knowledge a week later might seem highly implausible for children: it contrasts sharply with science curricula recommended for young children by even the most ambitious interpretations of the Next Generation Science Standards^30^. It also contradicts adults’ intuitions. To demonstrate this contradiction, we asked adults to estimate the earliest age at which a majority of children would understand a video about a variety of topics ranging from simple to complex.

**Fig. 1:**
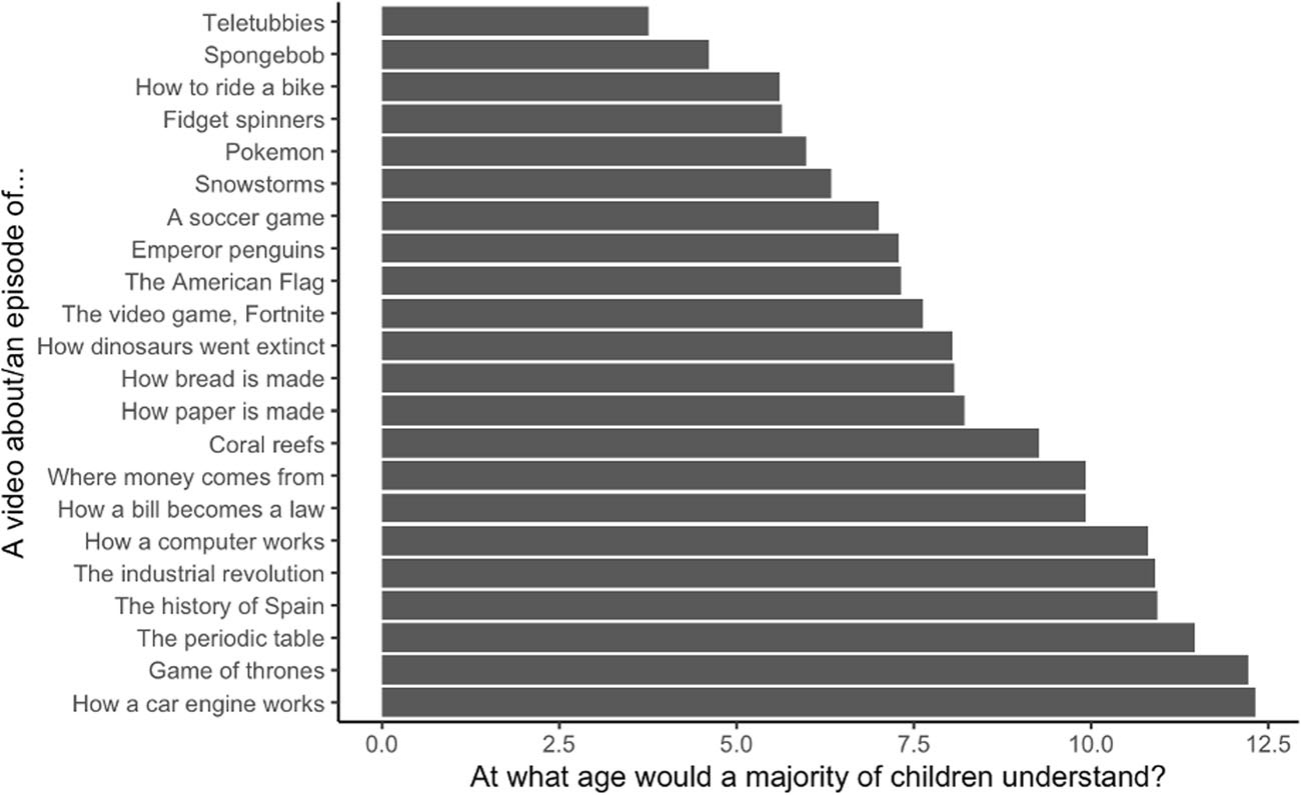
Adults’ average age of understanding judgements. Bars depict adults’ mean ratings at which age a majority of children would understand a video about a variety of topics.

We recruited 41 adults via Amazon Mechanical Turk who participated for pay. None were excluded. In total, 22 video topics were chosen that covered a broad range of content, including videos explicitly aimed at young children (e.g., Teletubbies) and adults (e.g., Game of Thrones), see Fig. 1 for a full list of items. Participants were shown a series of items and asked to rate “at what age a majority of children would understand a video about/an episode of [topic]” on a scale of 0–18 years. Participants repeated this procedure for each item, order-randomized. Of all 22 items, adults rated a video of how a car engine works as requiring the highest age to understand, at over 12 years (M = 12.32, SD = 3.57), eclipsing videos about other complex or adult-oriented topics such as the industrial revolution, Game of Thrones, and how a computer works.

However, young children’s sustained interest in mechanism suggests that adults’ pessimistic assessment may be misguided, especially given children’s impressive ability to evaluate others’ knowledge based on their causal understanding^31^. We therefore expected to find lasting cognitive benefits of presenting mechanistically complex events to young children. We tested children aged 6–9 years. While children of this age can reason about those who possess mechanistic knowledge, they have received minimal formal education on mechanisms and possess little mechanistic knowledge themselves^30^. Therefore, in addition to being well under the age at which adults expect them to be able to understand how a car engine works, 6–9-year-olds’ performance in the current study is less likely to be influenced by knowledge they already possess compared with older children and adults.

We predicted that children who viewed the video would remember mechanistic details, such as the names and movement of parts. We also expected their memory of those details to decay over time. Most critically, we predicted that children who viewed the video would acquire knowledge of relevant abstract causal patterns compared with control children who had not viewed the video, even though these patterns were never directly communicated to them. We further predicted that children would retain this abstract knowledge a week after watching the video.

## Results

See supplementary materials for a comprehensive analysis.

### Detailed mechanistic knowledge

To analyze children’s performance in the part names and movement tasks, we fitted a linear regression to the data; the number of items correct was the dependent variable, while condition (immediate test, delayed test, and control) and age (in months) were predictors. We used Bonferroni-adjusted post hoc comparisons to compare performance between conditions. Children in the immediate test condition (M = 4.37, SD = .82) remembered engine-part names marginally better than those in the delayed test condition (M = 4.02, SD = 1.00), *t*(174) = 2.08, *p* = *0.079*, who performed better than their counterparts in the control condition (M = 1.92, SD = 1.03), *t*(174) = 12.46, *p* < *0.001*. Memory for part names improved with age, *t*(174) = 3.21, *p* = *0.002*. In the part-movement task, memory was comparable in the immediate (M = 3.12, SD = 0.90) and delayed test conditions (M = 2.77, SD = 1.29), *t*(174) = 1.63, *p* = *0.21*, while delayed test participants performed better than those in the control condition (M = 1.95, SD = 1.32), *t*(174) = 3.81, *p* < *0.001*. Memory for part movement was consistent across ages, *t*(174) = 0.79, *p* = *0.43*.

### Abstracted causal knowledge

To analyze children’s general performance in the expert-detection task, we fitted a linear regression to the data; the number of items correct was the dependent variable, while condition and age were predictors. We used Bonferroni-adjusted post hoc comparisons to compare performance between conditions. Children in the immediate test condition (M = 4.07, SD = 1.21) were better at detecting car-engine experts relative to those in the delayed test condition (M = 3.5, SD = 1.11), *t*(174) = 2.43, *p* = *0.048*, who in turn were better than those in the control condition (M = 2.67, SD = 1.31), *t*(174) = 6.50, *p* < *0.001*. This pattern becomes more pronounced with age: as age increases, the difference in expert-detection performance between the immediate and delayed test conditions and control condition widens, *t*(174) = 4.37, *p* < *0.001*, and *t*(174) = 2.69, *p* = *.008*, respectively. Importantly, these patterns are driven by both better performance with age in the immediate and delayed test conditions and poorer performance with age in the control condition. This suggests that the younger children in our sample performed at or near chance across conditions, while older children identified the expert worse than chance at baseline and better than chance following the video.

To analyze children’s acquisition of specific abstract concepts, we fitted the data to a mixed-effects logistic-regression model. Expert choice (1 if correct, 0 if incorrect) was the dependent variable, while condition and abstract concept (synchronicity, decentralized control, and containment) were predictors; participants were fit as random intercepts. We generated 95% confidence intervals (CIs) via the effects package (Fox, 2003) to compare performance in each condition to each other. Because children’s performance on the task varied significantly with age, we generated two separate models: one fitted to the data from 6- to 7-year-olds and the other fitted to the data from 8- to 9-year-olds.

### Synchronicity

Across both age groups, children in the immediate test condition performed significantly better on synchronicity items than those in the control condition: predicted 95% CI [0.74, 0.93] compared to [0.43, 69] for 6, 7-year-olds, and [0.82, 0.97] compared with [0.34, 0.59] for 8, 9-year-olds. However, only 8, 9-year-olds performed significantly better in the delayed test condition than the control condition a week later: [0.65, 0.87] compared with [0.43, 0.69] for 6, 7 year-olds and [0.67, 0.87] compared with [0.34, 0.59] for 8, 9 year-olds.

### Decentralized control

In all, 8, 9 year-olds, but not 6, 7 year-olds, in the immediate condition performed significantly better on decentralized control items than those in the control condition: [0.32, 0.58] compared with [0.41, 0.66] for 6, 7 year-olds, and [0.54, 0.78] compared with [0.18, 0.41] for 8, 9 year-olds. Neither age group in the delayed test condition performed significantly better than controls: [0.22, 0.46] compared with [0.41, 0.66] for 6, 7 s, and [0.36, 0.61] compared with [0.18, 0.41] for 8, 9 s.

### Containment

**Fig. 2:**
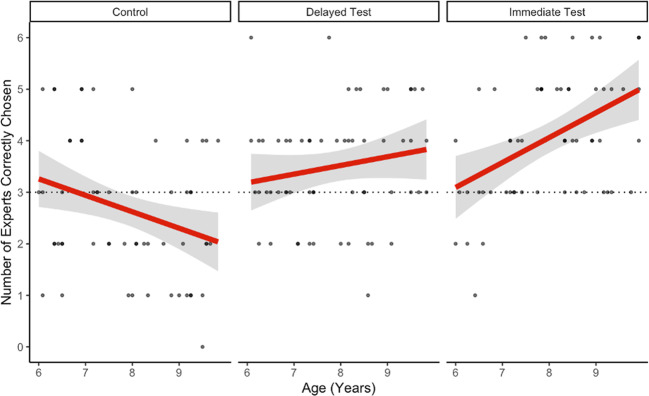
Expert-detection task performance by age and condition. Ribbons are 95% confidence intervals.

Neither age group in the immediate or delayed conditions performed significantly better on containment items compared with those in the control condition: [0.39, 0.65] and [0.44, 0.69] compared with [0.32, 0.58] for 6, 7 year-olds, and [0.49, 0.73] and [0.42, 0.67] compared with [0.28, 0.53] for 8, 9 year-olds (Fig. 2).

## Discussion

As predicted, children learned the names and movement of car-engine parts from the seven-minute video. However, contrary to our expectations, children remembered these details a week later, demonstrating impressive long-term retention of mechanistic details. Both immediately after viewing the video and a week later, children became better at detecting car-engine experts, with the older children in our sample showing the most improvement. Importantly, successfully choosing the expert in the current task relies on concepts implied but never explicitly mentioned in the video, suggesting that children retained an understanding of the underlying causal patterns in addition to mechanistic details. The current study tested three specific features: the containment of fluid, the synchronization of part movement, and the decentralized nature of how a car engine’s parts are controlled. All three depend on causal relations between engine subcomponents, and abstract away from particular relations and subcomponents to characterize how a car engine works as a whole.

Importantly, children in our sample did not uniformly acquire these abstract concepts. Children of all ages appeared to abstract synchronicity from the video, with older children retaining that knowledge a week later. Older, but not younger, children also abstracted decentralized control from the video, although that knowledge did not appear to persist after a week. Finally, there was not strong evidence that older or younger children acquired an abstract understanding of containment from the video at all. Future research is needed to uncover factors that make some causal abstractions more difficult to acquire than others.

Beyond being useful for understanding the mechanism at hand, how else might an understanding of causal patterns benefit learners? The expert-detection task itself suggests an enhanced ability to infer and evaluate the knowledge of others. Indeed, despite possessing little mechanistic knowledge themselves, children as young as six can use others’ mechanistic knowledge to distinguish between informants^21^ and make inferences about the limits of those informants’ knowledge^22^. An understanding of the causal mechanisms shared by classes of things, such as vehicles being propelled by engines, may guide these abilities.

The benefits of mechanistic exposure may also extend beyond the realm of adjudicating information sources. By helping learners focus on the most causally relevant patterns in a domain, mechanistic exposure could empower relearning of the same content or generalize to learning in the same high-level subject area^29^. This in turn could aid learners in grouping entities that share key causal patterns, even if they differ superficially^32,33^. In addition, an understanding of underlying causal patterns could support the categorization of new entities and the induction of newly learned properties to known ones based on their underlying causal features^34,35^. Future research could investigate how exposure to mechanistic details shapes children’s reasoning about concepts and categories beyond their ability to reason about expertise.

While mechanistic exposure clearly improved older children’s ability to detect expertise, several factors limit the generalizability and conclusions of the current study. First, because children also acquired concrete knowledge (e.g., about part names and movement) from the video, their subsequent improvement in expert detection might not be the result of causal abstractions formed while watching the video, but rather from their still-present concrete knowledge during the expert-detection task itself. While the first interpretation implies that children automatically abstracted causal patterns from mechanistic instruction, the second suggests they did so on an ad hoc basis when a need for such abstractions arose. Future research could tease these possibilities apart by using more complex mechanisms where concrete details are more difficult to remember, or by increasing the time duration between initial exposure and testing.

Second, the current study does not attempt to measure exactly how abstract children’s car-engine representations have become beyond the information presented in the video. The expert-detection task was designed not to repeat verbatim any of the language used in the video so that children who watched the video could not identify the car-engine expert purely due to verbal memory. However, some degree of verbal similarity between the video and the correct car engine expert was unavoidable (e.g., “squeeze the gas” vs “hold the gas in for a little bit”). In addition, the current study only measured the influence of mechanistic exposure on children’s ability to reason about others’ knowledge and expertise. Therefore, the granularity of children’s abstract causal knowledge and full range of benefits it provides also require further study. Nonetheless, prior work demonstrates that providing explanations to children or encouraging them to self-explain can focus their attention on causally relevant, as opposed to superficial, details of causal systems and improve their ability to intervene on them^18,19,36^. Future work could investigate how exposure to mechanistic explanations about a causal system influences children’s abilities to intervene on that system and on other systems with underlying similarities (e.g., steam engines). Such investigations would enable researchers to probe how abstract children’s mechanistic representations are, how they generalize to other systems, and what other abilities they support.

Third, the current study focuses on a single mechanical system, an internal combustion engine, and a small subset of causal patterns underlying its operation (e.g., synchronicity, containment, and decentralized control). The findings of the current study may therefore not smoothly generalize to other mechanisms, particularly those within other domains such as biology (e.g., how a kidney works) or economics (e.g., how inflation works) that children encounter in the classroom and elsewhere. Mechanistic exposure appears to cause children to reason differently about the complexity of human hearts^37^, although more systematic studies are needed to determine the precise abstractions involved. Nonetheless, the relatively short time span of the current intervention (around 7 min) suggests that more comprehensive interventions have the potential to more radically shift children’s intuitions.

Despite the perceived hurdles of learning how objects work, elaborate levels of mechanistic detail may nonetheless be pedagogically powerful. Exposure to a system’s inner workings provides insights into more general causal principles explaining how that system works or operates. Among other uses, these abstractions can be employed to evaluate the knowledge of others, even when one possesses little knowledge themselves. We find that children as young as eight who saw a video about how car engines work were reliably better able than controls to use abstract principles to detect “real” car-engine experts. The knowledge children acquired from a seven-minute video endured over time and influenced performance in a completely novel task a week later. Yet, the vast majority of adults surveyed thought the children we tested would be far too young to learn anything of importance from the video. In contrast, children not only learned a surprising amount, but were notably engaged. Out of the 120 children who watched the video, none elected to stop midway through, and many self-reported that they found the video to be interesting, often to their parents’ surprise.

We suggest that early exposure to complex causal systems can provide children with both concrete knowledge and abstract system-level knowledge deployable when reasoning about others’ knowledge. Instead of being overwhelmed and discouraged by the multitude of details, children are often fascinated by complex mechanisms. Yet, many classrooms set students up to fail by only assessing their memory of these details rather than the skills they enhance. These incomplete assessments of students’ abilities can give the impression that complex mechanisms are simply out of most learners’ reach^38^. Instead of sparing children encounters with these mechanisms altogether, we should recognize children’s early ability to take advantage of the flood of information with an eye for what they are actually gaining from it.

## Methods

### Participants

All research conducted was approved by the Yale University IRB and adhered to all ethical regulations regarding human subjects. In total, 180 children (*M*age = 95 months, range: 6:0–9:11, 96 males) participated in the initial study. Given the logistics of scheduling and completing two study sessions exactly one week apart, we determined that this was the largest feasible sample-size recruitment efforts could support. A post hoc power analysis, conducted via simulation using the SimR package in R^39^, confirmed that the current sample size had ample power to detect an effect of condition on expert-detection performance, 95% CI [0.92, 0.95]. Children took part via TheChildLab.com online platform^40^, where researchers engaged in online videoconferences with participants on a web-enabled device. The study stimuli were presented as a PowerPoint presentation shared within the videoconference. Participants were sampled from a pool of recruited families and completed two online sessions. Before the study sessions, families were provided with information about the study. In lieu of written consent, parents provided verbal consent for their child to participate, which was recorded; children also provided verbal assent during the study session. In total, 14 participants who failed to attend the second session were excluded with replacement. Estimated household incomes were obtained for each family based on their zip code. A broad distribution of income levels participated ($13,468–$200,001), with the mean income level ($72,545) being lower than the national average when the study was conducted ($89,930).

### Materials

To teach children how a car engine works, we modified a video designed for skilled-trade instruction at the adult level by dubbing over the video with mechanistic explanation and commentary about an internal combustion engine. To measure concrete knowledge about a car engine, we used screenshots of the inside of a car engine to test participants’ memory of the names and movement of individual parts (see supplementary materials).

To measure children’s abstract causal knowledge, we presented children with pairs of informants (blue- or green-colored silhouettes) who each provided a statement about how car engines work, after which children were asked to pick the car-engine expert. The stimuli tested their understanding of three core features of car engines: decentralized control, containment, and synchronicity. These features abstract away from specific pieces of information presented in the video and concern the operation of car engines in aggregate. Importantly, none of these features were ever explicitly mentioned in the video, although they could in principle be inferred from the descriptions provided in the video. Decentralized control refers to the absence of a central causal agent (e.g., a motherboard) that singularly controls each part within a car engine; instead, each part causes another part(s) to move in a decentralized fashion. Containment refers to the way that fluid moves and is manipulated inside car engines; gasoline and air are deliberately trapped and manipulated instead of moving freely throughout. Finally, synchronicity refers to the way car engine parts move in relation to each other. Instead of moving independently at different speeds and rates of change, car engine parts move in unison because they mutually determine each other’s movement. Two pairs of statements (one true, one false) were generated for each concept, yielding six pairs of statements in total. For each pair, one statement correctly described how a car-engine works (mean word count = 15), while the other described an opposing, yet intuitive alternative (mean word count = 17). Neither statement reused language from the car-engine video, see Table 1 for a full list of the expert-detection items.

**Table 1:** Expert detection task stimuli.

Abstract Concept	Expert	Non-expert
Decentralized Control (1)	In an engine, the speed of most parts is controlled by the parts right next to them.	In an engine, there is one part that is connected to most of the other parts and controls how fast they go.
Decentralized Control (2)	Each part in the engine makes a different part move.	There is one part in the engine that makes most of the other parts move.
Containment (1)	The valves are important because they hold the gas in for a little bit.	The valves are important because they clean the bad stuff out of the gas.
Containment (2)	For a car engine to work well, sometimes air needs to be trapped inside the engine.	For a car engine to work well, air has to flow in and out of the engine without stopping.
Synchronicity (1)	The belt is important because it helps the parts in the engine move together at the same speed.	The belt is important because it keeps the parts in place when the engine vibrates.
Synchronicity (2)	In an engine, when one part goes faster, all the other parts have to go faster too.	In an engine, when some parts go faster, other parts have to slow down to save energy.

Procedure: Sixty participants were each assigned to one of three conditions: immediate test, delayed test, or control^1^. The ages of the children in the three conditions did not differ (*ps* > *0.7*) The immediate-test condition measured acquisition of concrete and abstracted knowledge directly after watching the video. Children first viewed the video and afterward completed four sets of test questions.

In the understanding section, we first asked children “do you think the video was easy to understand or hard to understand,” followed by asking if it was kind of easy/hard or very easy/hard. We then asked children “how much do you think you learned from the video; do you think you learned a small amount or a large amount,” followed by asking if it was a kind of small/large amount or a very small/large amount.

In the part names section, children were shown a picture of a car engine with a given part highlighted. Below the image, the names of three parts were depicted in blue, yellow, and green, respectively, one name was correct, while the other two were car-part names not mentioned in the video. Children were told “here are three things this part could be called. Is it called the [name 1] in blue, the [name 2] in yellow, or the [name 3] in green?”. Children repeated this procedure for each of five parts (belt, pistons, camshaft, crank, and valves), order counterbalanced.

In the expert-detection section, children were told, “in this new activity, you’re going to hear two people say something about a car engine, but only one of them really knows about car engines and is a car engine expert. The other one is just guessing. So your job is to listen to what each person says and tell me which one you think is the real car engine expert.” Children were then shown pairs of silhouettes, one in blue and one in green. The experimenter then read a statement from each one (see Table 1) and asked children, “so who do you think is the real car engine expert, the blue one or the green one?”. This procedure was repeated for all six items.

In the part-movement section, children were asked about how individual parts within the engine moved. First, an experimenter told the child “I’m going to ask you about how the parts in a car engine move. It’s ok if you don’t remember, just try your best!” Children were shown an image of a car-engine part (the target), with three additional parts and their names in blue, green, and yellow squares below. Children were asked “which part makes the [target] move? Do you think it’s the [part in blue] in blue, the [part in yellow] in yellow, or the [part in green] in green that makes the [target] move?” Children repeated this procedure for four parts (camshaft, crank, valves, and belt), order counterbalanced.

The delayed-test condition measured children’s retention of detailed mechanistic information and abstracted causal patterns after one week. Participants in this condition watched the video and were then asked the first set of test questions (understanding). Then one week later, participants completed the part names, expert detection, and part-movement questions during a second online session.

In the control condition, participants completed one online session. This study measured children’s baseline performance, without viewing the video, on part names, expert detection, and part movement. We had initially planned on using a partial control condition in which children only completed the expert-detection task, but given children’s strong performance on the part names and movement tasks in the immediate and delayed-test conditions, we later switched to a complete control condition where participants completed the same measures as the two other conditions. Because children’s expert judgments were the same in both control conditions, we report here only the full control (data and analysis for the partial-control condition can be found in the supplementary materials).

### Reporting summary

Further information on research design is available in the Nature Research Reporting Summary linked to this article.

## Supplementary information

Supplementary Movie 1

Reporting Summary

Supplementary Information

## Data Availability

The datasets generated and analyzed in this paper are available in this OSF repository, https://osf.io/dtzxy/?view_only=ec0a1bab92bc46ac9cc9e9ab0548c9d9.

## Code availability

The code used to analyze the data and generate the images used in this paper is available in this OSF repository, https://osf.io/dtzxy/?view_only=ec0a1bab92bc46ac9cc9e9ab0548c9d9.
