# Supplementary material for: No guts, no glory: underestimating the benefits of providing children with mechanistic details
Source: NPJ Sci Learn. 2021 Oct 22;6:30. doi: 10.1038/s41539-021-00108-5 (PMC8536730; doi:10.1038/s41539-021-00108-5)
Supplement: Supplementary file 2 — Reporting Summary [file 41539_2021_108_MOESM2_ESM.pdf]

nature research

Corresponding author(s): Aaron Chuey
chuey@stanford.edu

Last updated by author(s): Aug 9, 2021

Reporting Summary

Nature Research wishes to improve the reproducibility of the work that we publish. This form provides structure for consistency and transparency in reporting. For further information on Nature Research policies, see our Editorial Policies and the Editorial Policy Checklist.

Statistics

For all statistical analyses, confirm that the following items are present in the figure legend, table legend, main text, or Methods section.

n/a	Confirmed	
☐	☒	The exact sample size (n) for each experimental group/condition, given as a discrete number and unit of measurement
☐	☒	A statement on whether measurements were taken from distinct samples or whether the same sample was measured repeatedly
☐	☒	The statistical test(s) used AND whether they are one- or two-sided<br>*Only common tests should be described solely by name; describe more complex techniques in the Methods section.*
☐	☒	A description of all covariates tested
☐	☒	A description of any assumptions or corrections, such as tests of normality and adjustment for multiple comparisons
☐	☒	A full description of the statistical parameters including central tendency (e.g. means) or other basic estimates (e.g. regression coefficient) AND variation (e.g. standard deviation) or associated estimates of uncertainty (e.g. confidence intervals)
☐	☒	For null hypothesis testing, the test statistic (e.g. F, t, r) with confidence intervals, effect sizes, degrees of freedom and P value noted<br>*Give P values as exact values whenever suitable.*
☒	☐	For Bayesian analysis, information on the choice of priors and Markov chain Monte Carlo settings
☒	☐	For hierarchical and complex designs, identification of the appropriate level for tests and full reporting of outcomes
☒	☐	Estimates of effect sizes (e.g. Cohen's d, Pearson's r), indicating how they were calculated

Our web collection on statistics for biologists contains articles on many of the points above.

Software and code

Policy information about availability of computer code

Data collection	Code was not used to collect data in the current study.
Data analysis	The data was analyzed in R using the base statistical functions as well as the lme4 and effects packages.

For manuscripts utilizing custom algorithms or software that are central to the research but not yet described in published literature, software must be made available to editors and reviewers. We strongly encourage code deposition in a community repository (e.g. GitHub). See the Nature Research guidelines for submitting code & software for further information.

Data

Policy information about availability of data

All manuscripts must include a data availability statement. This statement should provide the following information, where applicable:

- Accession codes, unique identifiers, or web links for publicly available datasets
- A list of figures that have associated raw data
- A description of any restrictions on data availability

All data, stimuli, and code related to the current studies will be made publicly available upon publication. Reviewers can view the repository at the following link: https://osf.io/dtzxy/?view_only=ec0a1bab92bc46ac9cc9e9ab0548c9d9

Field-specific reporting

Please select the one below that is the best fit for your research. If you are not sure, read the appropriate sections before making your selection.

☐ Life sciences ☒ Behavioural & social sciences ☐ Ecological, evolutionary & environmental sciences

For a reference copy of the document with all sections, see nature.com/documents/nr-reporting-summary-flat.pdf

Behavioural & social sciences study design

All studies must disclose on these points even when the disclosure is negative.

Study description	Participants were assigned to one of three conditions (immediate test, delayed test, control) between-subjects. Participants in the immediate test condition viewed a video about how a car engine work and completed three main tasks: part names, expert detection, and part movement. In the part names task, participants were shown series of parts and asked to select which of three options the part was called. In the expert detection task, participants were shown a 6 pairs of individuals who each provided a statement. For each pair, children were asked which was a car engine expert. For the part movement task, participants were shown a series of 4 car engine parts. For each, they were asked which other part among three made that part move. Participants in the delayed test condition watched the video and completed the part names, expert detection, and part movement tasks a week later. Participants in the control condition did not watch the video, and completed the part names, expert detection, and part movement tasks.
Research sample	180 children (Mage = 95 months, range: 6:0 to 9:11, 96 males) participated in the initial study. Children took part via TheChildLab.com online platform (Sheskin & Keil, 2018), where researchers engaged in online videoconferences with participants on a web-enabled device. 14 participants who failed to attend the second session were excluded with replacement. Estimated household incomes were obtained for each family based on their zip code. A broad distribution of income levels participated ($13,468 - $200,001), with the mean income level ($72, 545) being lower than the national average ($89,930).
Sampling strategy	Participants were sampled randomly from families who signed up to participate on TheChildLab.com. Families were advertised to primarily via Facebook advertising, Google advertising, and word of mouth. Sample size was set at 30 per age age group (6-7, and 8-9) per condition yielding 180 participants total. Given the logistics of scheduling and completing two study sessions exactly one week apart, we determined that this was the largest feasible sample size recruitment efforts could support.
Data collection	Participants in the immediate, delayed test, and expert-only control conditions signed up to participate for two sessions approximately one week apart. However, given children's strong performance on the part names and movement tasks, we added a comprehensive control condition. Participants assigned to this condition only signed up for a single session. However, data in the expert-only and comprehensive control conditions was found not to differ significantly, so only data from the comprehensive control condition is reported in the manuscript. The study was conducted via Adobe Connect Video conferencing software. Participant's verbal responses were coded real-time by the experimenter and were later reviewed based on the participant's recorded audio. Only the experimenter, participant, and participant's parent/guardian were present during the study session. Because the experiment was methodologically and logistically complex, only two experimenters were trained to administer the study. The study design did not allow for blinding by condition, and neither experimenters were blind to the research hypothesis.
Timing	Data collection for the immediate test, delayed test, and expert-only control conditions took place from approximately September 2017-December 2018. Data collection for the comprehensive control condition took place from approximately January 2019-January 2020.
Data exclusions	14 participants who failed to attend the second study session were excluded with replacement. No others were excluded for any reason.
Non-participation	No participants dropped out of the study or declined to participate during a study session. Although, as stated above, 14 participants who failed to attend the second study session were excluded with replacement.
Randomization	Participants were randomly assigned to condition for the immediate test, delayed test, and expert-only control conditions. Because data collection took place later for the comprehensive control condition, condition assignment was not randomly, although participants were randomly sampled from the same subject pool.

Reporting for specific materials, systems and methods

We require information from authors about some types of materials, experimental systems and methods used in many studies. Here, indicate whether each material, system or method listed is relevant to your study. If you are not sure if a list item applies to your research, read the appropriate section before selecting a response.

Materials & experimental systems

n/a	Involved in the study	
☒	☐	Antibodies
☒	☐	Eukaryotic cell lines
☒	☐	Palaeontology and archaeology
☒	☐	Animals and other organisms
☐	☒	Human research participants
☒	☐	Clinical data
☒	☐	Dual use research of concern

Methods

n/a	Involved in the study	
☒	☐	ChIP-seq
☒	☐	Flow cytometry
☒	☐	MRI-based neuroimaging

Human research participants

Policy information about studies involving human research participants

Population characteristics	See above
Recruitment	Participants were sampled randomly from families who signed up to participate on TheChildLab.com. Families were advertised to primarily via Facebook advertising, Google advertising, and word of mouth. Although this does require participants to have internet access, a broad distribution of income levels participated ($13,468 - $200,001), with the mean income level ($72, 545) being lower than the national average ($89,930). Additionally, because participants had to sign up for two sessions, this could have biased the sample to those who have more time or interest in participating.
Ethics oversight	All research conducted was approved by the Yale University IRB

Note that full information on the approval of the study protocol must also be provided in the manuscript.
