# Supplementary material for: No guts, no glory: underestimating the benefits of providing children with mechanistic details
Source: NPJ Sci Learn. 2021 Oct 22;6:30. doi: 10.1038/s41539-021-00108-5 (PMC8536730; doi:10.1038/s41539-021-00108-5)

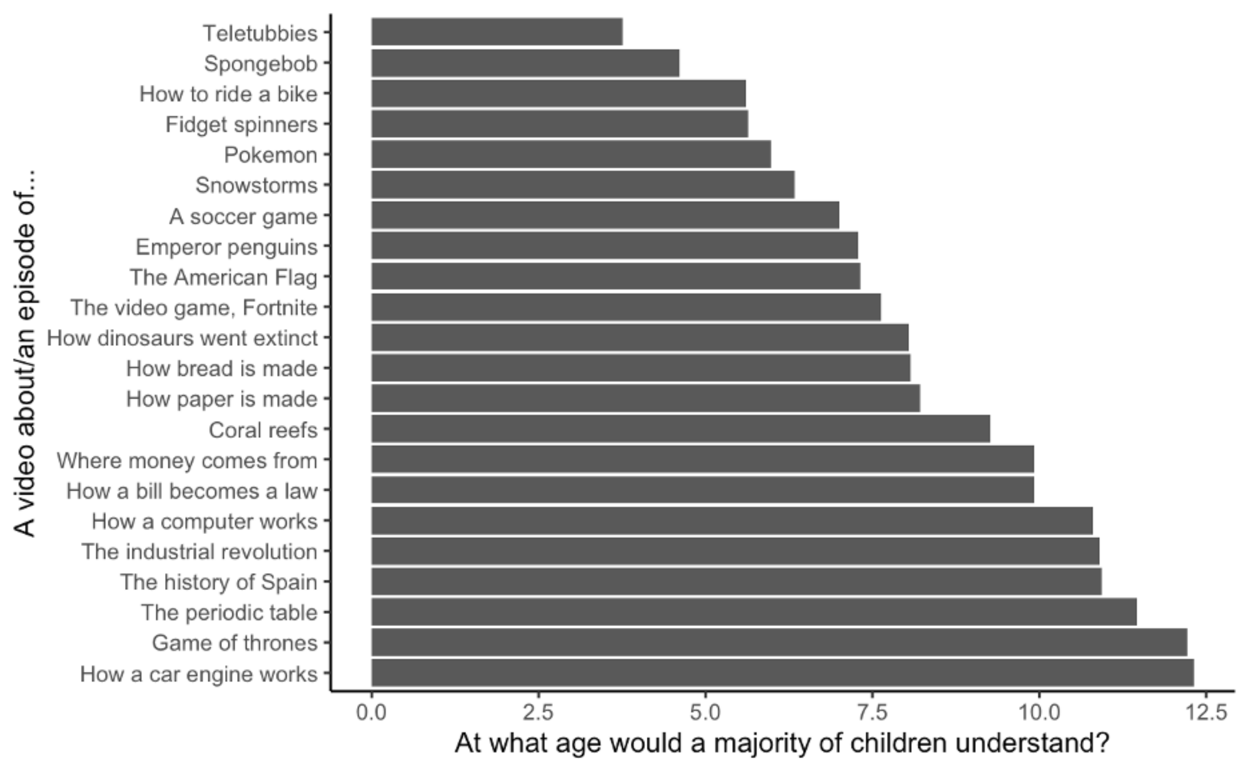

Teletubbies
Spongebob
How to ride a bike
Fidget spinners
Pokemon
Snowstorms
A soccer game
Emperor penguins
The American Flag
The video game, Fortnite
How dinosaurs went extinct
How bread is made
How paper is made
Coral reefs
Where money comes from
How a bill becomes a law
How a computer works
The industrial revolution
The history of Spain
The periodic table
Game of thrones
How a car engine works
A video about/an episode of...
0.0
2.5
5.0
7.5
10.0
12.5
At what age would a majority of children understand?

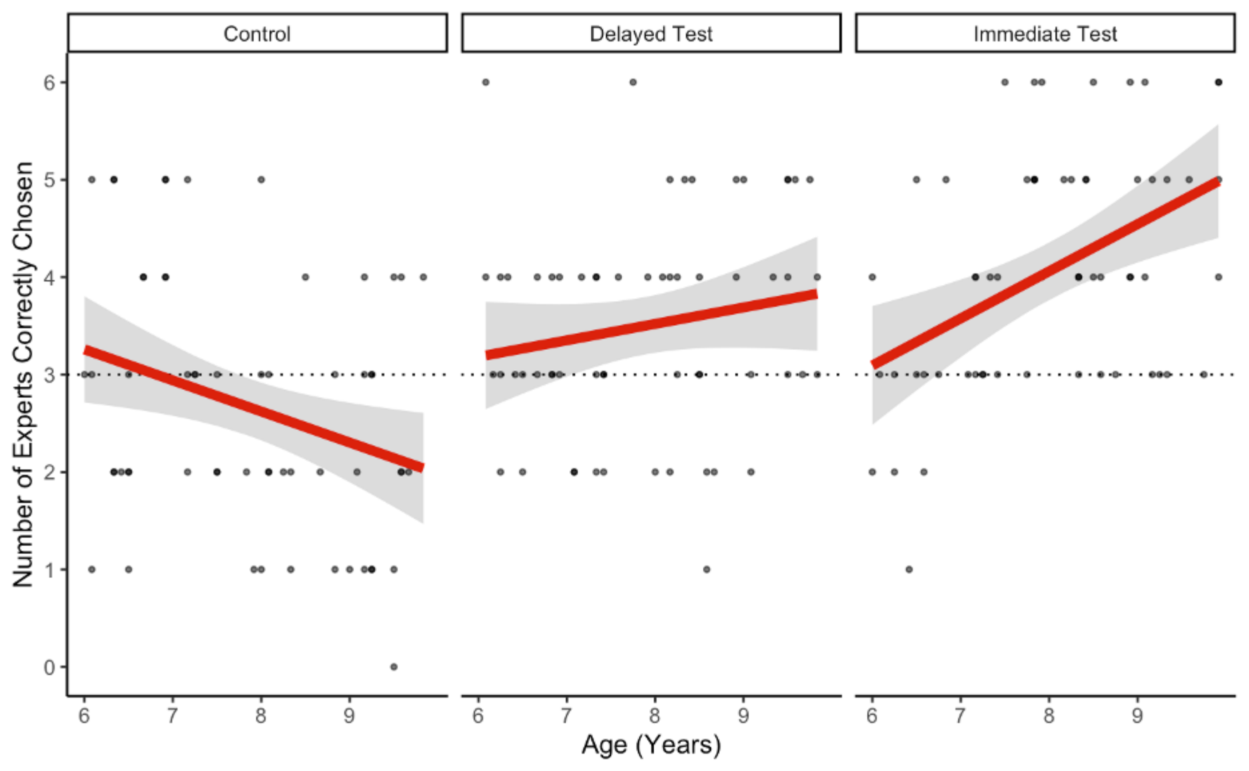
Control
Delayed Test
Immediate Test
Number of Experts Correctly Chosen
0
1
2
3
4
5
6
6
7
8
9
Age (Years)

Abstract Concept	Expert	Non-expert
Decentralized Control (1)	In an engine, the speed of most parts is controlled by the parts right next to them.	In an engine, there is one part that is connected to most of the other parts and controls how fast they go.
Decentralized Control (2)	Each part in the engine makes a different part move.	There is one part in the engine that makes most of the other parts move.
Containment (1)	The valves are important because they hold the gas in for a little bit.	The valves are important because they clean the bad stuff out of the gas.
Containment (2)	For a car engine to work well, sometimes air needs to be trapped inside the engine.	For a car engine to work well, air has to flow in and out of the engine without stopping.
Synchronicity (1)	The belt is important because it helps the parts in the engine move together at the same speed.	The belt is important because it keeps the parts in place when the engine vibrates.
Synchronicity (2)	In an engine, when one part goes faster, all the other parts have to go faster too.	In an engine, when some parts go faster, other parts have to slow down to save energy.

[Figure Legends]

Fig 1. Adult's mean ratings at which age a majority of children would understand a video about a variety of topics

Table 1. Expert detection task stimuli

Fig 2. Expert detection task performance by age and condition. Ribbons are 95% confidence intervals.

Supplementary Video 1. Video used in the experiment that explains how an internal combustion engine works. Video was modified and used with permission from Autotechlabs [Source Video ID: DKF5dKo_r_Y].

Full Analysis

Aaron Chuey

2021-04-07 18:04:42

- 1 Data Preprocessing
- 2 Summary of measures
- 3 Analysis of concrete knowledge
- 4 Aggregated Analysis of abstract knowledge
- 5 Fine Grained Analysis of Abstract Knowledge
- 6 Power Analysis
- 7 Adult Study
- 8 Population Statistics

1 Data Preprocessing

Load in libraries

```
library("knitr") # for knitting things
library("tidyverse") # for all things tidyverse
```

```
## Warning: package 'tibble' was built under R version 3.6.2
```

```
library("emmeans") # for planned contrasts
library("plotrix") # for error bars
library("lme4") # for mixed effects logistic regression
library("effects") # for model predictions
library("simr") # for power analysis
# these options here change the formatting of how comments are rendered
opts_chunk$set(
  comment = "",
  results = "hold",
  fig.show = "hold")

# set the default ggplot theme
theme_set(theme_classic())

# include references for used packages
knitr::write_bib(.packages(), "references/packages.bib")
```

Preprocess data

```
#read in data file
df.engine = read_csv("data/ENG_data.csv")

#factor Condition levels, rename age groups, calculate age in months
df.engine = df.engine %>%
  mutate(Condition = factor(Condition,
                            levels = c("F", "C", "D", "I"),
                            labels = c("Control", "Expert Only Control", "Delayed Test", "Immediate Test")
                            )
         ) %>%
  mutate(Age_Group = as.character(Age_Group)) %>%
  mutate(Age_Group = ifelse(Age_Group == 1, "6-7", "8-9")) %>%
  separate(Age, c("years", "months"), ";") %>%
  mutate(Age = as.numeric(years)*12 + as.numeric(months))
```

2 Summary of measures

Mean and Standard Deviation for Number of Items Correct in Part Names, Part Movement, and Expert Detection Tasks By Condition

```
df.summary = df.engine %>%
  filter(Condition != "Expert Only Control") %>%
  dplyr::group_by(Condition) %>%
  dplyr::summarize(Part.Names = mean(Parts_Score),
            sd.Part.Name = sd(Parts_Score),
            Part.Movement = mean(Movement_Score),
            sd.Part.Movement = sd(Movement_Score),
            Expert.Detection = mean(Expert_Score),
            sd.Expert.Detection = sd(Expert_Score))
df.summary
```

```
# A tibble: 3 x 7
  Condition Part.Names sd.Part.Name Part.Movement sd.Part.Movement
  <fct>          <dbl>        <dbl>         <dbl>            <dbl>
1 Control         1.92        1.03           1.95            1.32
2 Delayed …       4.02        1.00           2.77            1.29
3 Immediat…       4.37        0.823          3.12            0.904
# … with 2 more variables: Expert.Detection <dbl>, sd.Expert.Detection <dbl>
```

3 Analysis of concrete knowledge

Linear Regression predicting part names score based on condition (immediate, delayed, and full control), age (months), and age by condition interaction

```
lm_pn = lm(Parts_Score ~ Condition*Age, df.engine %>% filter(Condition != "Expert Only Control"))
lm_pn %>%
  summary()
```

```

Call:
lm(formula = Parts_Score ~ Condition * Age, data = df.engine %>%
    filter(Condition != "Expert Only Control"))

Residuals:
     Min       1Q   Median       3Q      Max
-3.01715 -0.70389 -0.01583  0.68976  2.01138

Coefficients:
                            Estimate Std. Error t value Pr(>|t|)
(Intercept)                -0.580559   0.788011  -0.737  0.46227
ConditionDelayed Test       4.593552   1.143413   4.017 8.74e-05 ***
ConditionImmediate Test     2.498614   1.167473   2.140  0.03373 *
Age                         0.026486   0.008263   3.205  0.00161 **
ConditionDelayed Test:Age  -0.026448   0.011978  -2.208  0.02855 *
ConditionImmediate Test:Age -0.001024   0.012125  -0.084  0.93278
---
Signif. codes:  0 '***' 0.001 '**' 0.01 '*' 0.05 '.' 0.1 ' ' 1

Residual standard error: 0.9159 on 174 degrees of freedom
Multiple R-squared:  0.6078,    Adjusted R-squared:  0.5965
F-statistic: 53.93 on 5 and 174 DF,  p-value: < 2.2e-16
```

Planned contrasts comparing part names score in the immediate test to the delayed test condition and in the delayed test condition to the comprehensive control condition.

```
contrasts = list(delayed_to_control = c(-1, 1, 0),
                 immediate_to_delayed = c(0, -1, 1))
leastsquare = emmeans(lm_pn, "Condition")
```

```
NOTE: Results may be misleading due to involvement in interactions
```

```
contrast(leastsquare,
         contrasts,
         adjust = "bonferroni")
```

```
 contrast             estimate    SE  df t.ratio p.value
 delayed_to_control       2.08 0.167 174 12.433  <.0001
 immediate_to_delayed     0.32 0.168 174  1.912  0.1150

P value adjustment: bonferroni method for 2 tests
```

Part names score by condition and age group; error bars represent standard errors

```
engine.pred_name = predict(lm_pn, df.engine %>% filter(Condition != "Expert Only Control"), interval = "con

df.engine_namefit = df.engine %>%
  filter(Condition != "Expert Only Control") %>%
  mutate(fit = engine.pred_name[,"fit"],
         lwr = engine.pred_name[,"lwr"],
         upr = engine.pred_name[,"upr"])

ggplot(data=df.engine_namefit,
       aes(x = Age,
           y = Parts_Score)
       ) +
  geom_point(alpha = .6,
             size = .8) +
  geom_hline(yintercept=c(5/3),
             linetype="dotted") +
  labs(x ="Age (Years)",
       y = "Number of Part Name Items Correct") +
  scale_x_continuous(limits = c(72, 119),
                     breaks = c(72, 84, 96, 108),
                     labels = c("6", "7", "8", "9")) +
  scale_y_continuous(limits = c(0, 5),
                     breaks = c(0, 1, 2, 3, 4, 5)) +
  facet_wrap(~Condition) +
  geom_line(aes(x = Age, y = fit),
            col = "red", size = 2) +
  geom_ribbon(aes(x = Age,
                  ymin = lwr,
                  ymax = upr),
              alpha = .2)
```

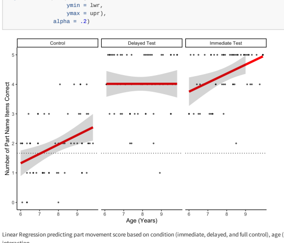

Linear Regression predicting part movement score based on condition (immediate, delayed, and full control), age (months), and age by condition interaction

```
lm_pm = lm(Movement_Score ~ Condition*Age, df.engine %>% filter(Condition != "Expert Only Control"))
lm_pm %>%
  summary()
```

```

Call:
lm(formula = Movement_Score ~ Condition * Age, data = df.engine %>%
    filter(Condition != "Expert Only Control"))

Residuals:
    Min      1Q  Median      3Q     Max
-2.7348 -0.8150  0.1329  0.8570  2.2264

Coefficients:
                            Estimate Std. Error t value Pr(>|t|)
(Intercept)                  1.16841    1.00422   1.164    0.246
ConditionDelayed Test       -0.32527    1.45714  -0.223    0.824
ConditionImmediate Test     -0.55901    1.48780  -0.376    0.708
Age                          0.00829    0.01053   0.787    0.432
ConditionDelayed Test:Age    0.01205    0.01527   0.789    0.431
ConditionImmediate Test:Age  0.01778    0.01545   1.151    0.251

Residual standard error: 1.167 on 174 degrees of freedom
Multiple R-squared:  0.1903,    Adjusted R-squared:  0.167
F-statistic: 8.179 on 5 and 174 DF,  p-value: 5.881e-07
```

Planned contrast comparing part movement scores in the immediate and delayed conditions to full control condition, and in the immediate to delayed condition

```
contrasts = list(delayed_to_control = c(-1, 1, 0),
                 immediate_to_delayed = c(0, -1, 1))
leastsquare = emmeans(lm_pm, "Condition")
```

```
NOTE: Results may be misleading due to involvement in interactions
```

```
contrast(leastsquare,
         contrasts,
         adjust = "bonferroni")
```

```
 contrast             estimate    SE  df t.ratio p.value
 delayed_to_control      0.820 0.213 174 3.843   0.0003
 immediate_to_delayed    0.311 0.214 174 1.455   0.2948

P value adjustment: bonferroni method for 2 tests
```

Part movement score by condition and age

```
engine.pred_mov = predict(lm_pm, df.engine %>% filter(Condition != "Expert Only Control"), interval = "conf

df.engine_movefit = df.engine %>%
  filter(Condition != "Expert Only Control") %>%
  mutate(fit = engine.pred_mov[,"fit"],
         lwr = engine.pred_mov[,"lwr"],
         upr = engine.pred_mov[,"upr"])

ggplot(data=df.engine_movefit,
       aes(x = Age,
           y = Movement_Score)
       ) +
  geom_point(alpha = .6,
             size = .8) +
  geom_hline(yintercept=c(4/3),
             linetype="dotted") +
  labs(x ="Age (Years)",
       y = "Number of Part Movement Items Correct") +
  scale_x_continuous(limits = c(72, 119),
                     breaks = c(72, 84, 96, 108),
                     labels = c("6", "7", "8", "9")) +
  scale_y_continuous(limits = c(0, 4),
                     breaks = c(0, 1, 2, 3, 4)) +
  facet_wrap(~Condition) +
  geom_line(aes(x = Age, y = fit),
            col = "red", size = 2) +
  geom_ribbon(aes(x = Age,
                  ymin = lwr,
                  ymax = upr),
              alpha = .2)
```

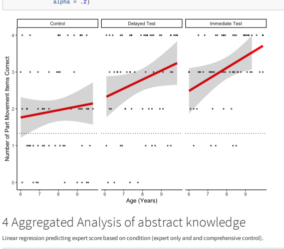

4 Aggregated Analysis of abstract knowledge

Linear regression predicting expert score based on condition (expert only and and comprehensive control).

```
lm_es_control = lm(Expert_Score ~ Condition,
                   df.engine %>%
                     filter(Condition == "Control" | Condition == "Expert Only Control"))
lm_es_control %>%
  summary()
```

```

Call:
lm(formula = Expert_Score ~ Condition, data = df.engine %>% filter(Condition ==
    "Control" | Condition == "Expert Only Control"))

Residuals:
    Min      1Q  Median      3Q     Max
-2.7333 -0.7333  0.2667  1.2667  2.3333

Coefficients:
                             Estimate Std. Error t value Pr(>|t|)
(Intercept)                   2.66667    0.16936  15.746   <2e-16 ***
ConditionExpert Only Control  0.06667    0.23951   0.278    0.781
---
Signif. codes:  0 '***' 0.001 '**' 0.01 '*' 0.05 '.' 0.1 ' ' 1

Residual standard error: 1.312 on 118 degrees of freedom
Multiple R-squared:  0.0006562, Adjusted R-squared:  -0.007813
F-statistic: 0.07748 on 1 and 118 DF,  p-value: 0.7812
```

Linear regression predicting expert score based on condition (immediate, delayed, and full control), age, and age by condition interaction

```
lm_es = lm(Expert_Score ~ Condition*Age, df.engine %>% filter(Condition != "Expert Only Control"))
lm_es %>%
  summary()
```

```

Call:
lm(formula = Expert_Score ~ Condition * Age, data = df.engine %>%
    filter(Condition != "Expert Only Control"))

Residuals:
    Min      1Q  Median      3Q     Max
-2.6187 -0.7857 -0.1249  0.7839  2.8035

Coefficients:
                            Estimate Std. Error t value Pr(>|t|)
(Intercept)                  5.17093    0.99321   5.206 5.39e-07 ***
ConditionDelayed Test       -3.00169    1.44116  -2.083 0.038729 *
ConditionImmediate Test     -4.97872    1.47148  -3.383 0.000884 ***
Age                         -0.02656    0.01042  -2.550 0.011626 *
ConditionDelayed Test:Age    0.04063    0.01510   2.691 0.007809 **
ConditionImmediate Test:Age  0.06685    0.01528   4.374 2.09e-05 ***
---
Signif. codes:  0 '***' 0.001 '**' 0.01 '*' 0.05 '.' 0.1 ' ' 1

Residual standard error: 1.154 on 174 degrees of freedom
Multiple R-squared:  0.2744,    Adjusted R-squared:  0.2535
F-statistic: 13.16 on 5 and 174 DF,  p-value: 7.139e-11
```

Planned contrast comparing expert score in the immediate and delayed conditions, and delayed and full control conditions for all ages.

```
contrasts = list(immediate_to_delayed = c(0, -1, 1),
                 delayed_to_control = c(-1, 1, 0),
                 immediate_to_control = c(-1, 0, 1))
leastsquare = emmeans(lm_es, "Condition")
```

```
NOTE: Results may be misleading due to involvement in interactions
```

```
contrast(leastsquare,
         contrasts,
         adjust = "bonferroni")
```

```
 contrast             estimate    SE  df t.ratio p.value
 immediate_to_delayed    0.514 0.211 174 2.432   0.0481
 delayed_to_control      0.859 0.211 174 4.070   0.0002
 immediate_to_control    1.372 0.211 174 6.495   <.0001

P value adjustment: bonferroni method for 3 tests
```

Expert score by condition and age; error bars represent standard errors

```
engine.pred_exp = predict(lm_es, df.engine %>% filter(Condition != "Expert Only Control"), interval = "conf
df.engine_expertfit = df.engine %>%
  filter(Condition != "Expert Only Control") %>%
  mutate(fit = engine.pred_exp[,"fit"],
         lwr = engine.pred_exp[,"lwr"],
         upr = engine.pred_exp[,"upr"])

ggplot(data=df.engine_expertfit,
       aes(x = Age,
           y = Expert_Score)
       ) +
  geom_point(alpha = .6,
             size = .8) +
  geom_hline(yintercept=c(3),
             linetype="dotted") +
  labs(x ="Age (Years)",
       y = "Number of Experts Correctly Chosen") +
  scale_x_continuous(limits = c(72, 119),
                     breaks = c(72, 84, 96, 108),
                     labels = c("6", "7", "8", "9")) +
  scale_y_continuous(limits = c(0, 6),
                     breaks = c(0, 1, 2, 3, 4, 5, 6)) +
  facet_wrap(~Condition) +
  geom_line(aes(x = Age, y = fit),
            col = "red", size = 2) +
  geom_ribbon(aes(x = Age,
                  ymin = lwr,
                  ymax = upr),
              alpha = .2)
```

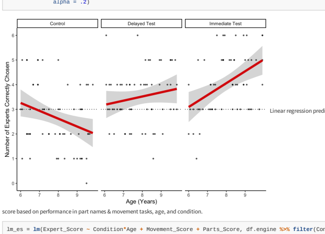

Linear regression predicting expert score based on performance in part names & movement tasks, age, and condition.

```
lm_es = lm(Expert_Score ~ Condition*Age + Movement_Score + Parts_Score, df.engine %>% filter(Condition != "
lm_es %>%
  summary()
```

```

Call:
lm(formula = Expert_Score ~ Condition * Age + Movement_Score +
    Parts_Score, data = df.engine %>% filter(Condition != "Expert Only Control"))

Residuals:
    Min      1Q  Median      3Q     Max
-2.4259 -0.8436 -0.1611  0.8666  2.7602

Coefficients:
                            Estimate Std. Error t value Pr(>|t|)
(Intercept)                  5.34215    0.99096   5.391 2.29e-07 ***
ConditionDelayed Test       -4.04165    1.49865  -2.697 0.007696 **
ConditionImmediate Test     -5.55788    1.48048  -3.754 0.000238 ***
Age                         -0.03220    0.01062  -3.032 0.002803 **
Movement_Score              -0.03529    0.07663  -0.461 0.645689
Parts_Score                  0.22390    0.09765   2.293 0.023067 *
ConditionDelayed Test:Age    0.04698    0.01525   3.082 0.002398 **
ConditionImmediate Test:Age  0.06771    0.01520   4.453 1.52e-05 ***
---
Signif. codes:  0 '***' 0.001 '**' 0.01 '*' 0.05 '.' 0.1 ' ' 1

Residual standard error: 1.144 on 172 degrees of freedom
Multiple R-squared:  0.2959,    Adjusted R-squared:  0.2673
F-statistic: 10.33 on 7 and 172 DF,  p-value: 8.681e-11
```

Expert detection preprocessing

```
df.expert = df.engine %>%
  pivot_longer(cols = c("CL1", "CL2", "CN1", "CN2", "SY1", "SY2"), names_to = "expert_item", values_to = "e
  mutate(expert_type = case_when(
    expert_item %in% c("CL1","CL2") ~ "Control",
    expert_item %in% c("CN1","CN2") ~ "Containment",
    expert_item %in% c("SY1", "SY2") ~ "Synchronicity"
  ))
df.expert
```

```
# A tibble: 1,440 x 21
   Participant years months Age_Group Gender Experimenter Condition    CB Order
         <dbl> <chr> <chr>  <chr>     <chr>  <chr>        <fct>     <dbl> <dbl>
 1           9 7     1      6-7       m      A            Expert O…     1     1
 2           9 7     1      6-7       m      A            Expert O…     1     1
 3           9 7     1      6-7       m      A            Expert O…     1     1
 4           9 7     1      6-7       m      A            Expert O…     1     1
 5           9 7     1      6-7       m      A            Expert O…     1     1
 6           9 7     1      6-7       m      A            Expert O…     1     1
 7          10 7     0      6-7       f      A            Expert O…     1     2
 8          10 7     0      6-7       f      A            Expert O…     1     2
 9          10 7     0      6-7       f      A            Expert O…     1     2
10          10 7     0      6-7       f      A            Expert O…     1     2
# … with 1,430 more rows, and 12 more variables: Understanding <dbl>,
#   Learning <dbl>, Parts_Score <dbl>, Movement_Score <dbl>, CL_Score <dbl>,
#   CN_Score <dbl>, SY_Score <dbl>, Expert_Score <dbl>, Age <dbl>,
#   expert_item <chr>, expert_correct <dbl>, expert_type <chr>
```

5 Fine Grained Analysis of Abstract Knowledge

Mixed effects logistic regression predicting expert score based on condition and abstract concept with random intercepts fitted by participant - All ages

```
log.model <- glmer(expert_correct ~ 1 + Condition*expert_type + (1 | Participant),
                   data = df.expert %>% filter(Condition != "Expert Only Control"),
                   family = binomial(link = "logit"))
log.model %>%
  summary()

# Calculating odds ratios
broom.mixed::tidy(log.model, exponentiate=TRUE, conf.int=TRUE) %>%
  mutate_if(is.numeric, round, 9)
```

```
Registered S3 method overwritten by 'broom.mixed':
  method      from
  tidy.gamlss broom
```

```
# Predicted effects
log.effects <- allEffects(log.model)
log.effects.df <- lapply(log.effects, as.data.frame)
log.effects.df
```

```
Generalized linear mixed model fit by maximum likelihood (Laplace
  Approximation) [glmerMod]
 Family: binomial  ( logit )
Formula: expert_correct ~ 1 + Condition * expert_type + (1 | Participant)
   Data: df.expert %>% filter(Condition != "Expert Only Control")

     AIC      BIC   logLik deviance df.resid
  1375.6   1425.5   -677.8   1355.6     1070

Scaled residuals:
    Min      1Q  Median      3Q     Max
-2.8155 -0.8673  0.3648  0.8664  1.3336

Random effects:
 Groups      Name        Variance Std.Dev.
 Participant (Intercept) 0.06235  0.2497
Number of obs: 1080, groups:  Participant, 141

Fixed effects:
                                             Estimate Std. Error z value
(Intercept)                                  -0.31065    0.18910  -1.643
ConditionDelayed Test                         0.58738    0.26629   2.206
ConditionImmediate Test                       0.66055    0.26751   2.469
expert_typeControl                           -0.10477    0.26435  -0.396
expert_typeSynchronicity                      0.34086    0.26181   1.302
ConditionDelayed Test:expert_typeControl     -0.57929    0.37376  -1.550
ConditionImmediate Test:expert_typeControl    0.03551    0.37304   0.095
ConditionDelayed Test:expert_typeSynchronicity    0.68940    0.39072   1.764
ConditionImmediate Test:expert_typeSynchronicity  1.36569    0.43009   3.175
                                             Pr(>|z|)
(Intercept)                                    0.1004
ConditionDelayed Test                          0.0274 *
ConditionImmediate Test                        0.0135 *
expert_typeControl                             0.6919
expert_typeSynchronicity                       0.1929
ConditionDelayed Test:expert_typeControl       0.1212
ConditionImmediate Test:expert_typeControl     0.9242
ConditionDelayed Test:expert_typeSynchronicity    0.0777 .
ConditionImmediate Test:expert_typeSynchronicity  0.0015 **
---
Signif. codes:  0 '***' 0.001 '**' 0.01 '*' 0.05 '.' 0.1 ' ' 1

Correlation of Fixed Effects:
           (Intr) CndtDT CndtIT expr_C expr_S CDT:_C CIT:_C CDT:_S
CndtnDlydTs -0.706
CndtnImmdtT -0.704  0.499
exprt_typCn -0.692  0.491  0.489
exprt_typSy -0.701  0.499  0.497  0.500
CndtnDTs:_C  0.492 -0.696 -0.349 -0.707 -0.355
CndtnITs:_C  0.491 -0.348 -0.696 -0.709 -0.355  0.501
CndtnDTs:_S  0.467 -0.662 -0.329 -0.335 -0.669  0.472  0.238
CndtnITs:_S  0.423 -0.299 -0.599 -0.305 -0.607  0.214  0.433  0.409
# A tibble: 10 x 9
   effect group term    estimate std.error statistic  p.value conf.low conf.high
   <chr>  <chr> <chr>      <dbl>     <dbl>     <dbl>    <dbl>    <dbl>     <dbl>
 1 fixed  <NA>  (Inte…     0.733     0.139    -1.64    0.100     0.506      1.06
 2 fixed  <NA>  Condi…     1.80      0.479     2.21    0.0274    1.07       3.03
 3 fixed  <NA>  Condi…     1.94      0.518     2.47    0.0135    1.15       3.27
 4 fixed  <NA>  exper…     0.901     0.238    -0.396   0.692     0.536      1.51
 5 fixed  <NA>  exper…     1.41      0.368     1.30    0.193     0.842      2.35
 6 fixed  <NA>  Condi…     0.560     0.209    -1.55    0.121     0.269      1.17
 7 fixed  <NA>  Condi…     1.04      0.387     0.0952  0.924     0.499      2.15
 8 fixed  <NA>  Condi…     1.99      0.779     1.76    0.0777    0.926      4.29
 9 fixed  <NA>  Condi…     3.92      1.69      3.18    0.00150   1.69       9.10
10 ran_pa… Part… sd__(…    0.250    NA        NA      NA        NA        NA
$`Condition:expert_type`
       Condition   expert_type       fit         se     lower     upper
1        Control   Containment 0.4229551 0.04615373 0.3359728 0.5149917
2   Delayed Test   Containment 0.5687427 0.04626420 0.4767744 0.6562023
3 Immediate Test   Containment 0.5865929 0.04608358 0.4943598 0.6731261
4        Control       Control 0.3976121 0.04571500 0.3122738 0.4896667
5   Delayed Test       Control 0.3995506 0.04568977 0.3141896 0.4914829
6 Immediate Test       Control 0.5697028 0.04633059 0.4775687 0.6572499
7        Control Synchronicity 0.5075520 0.04669002 0.4168018 0.5978072
8   Delayed Test Synchronicity 0.7870087 0.03799083 0.7032413 0.8521035
9 Immediate Test Synchronicity 0.8865977 0.02914567 0.8158208 0.9324289
```

Expert Detetion Performance by Condition and Abstract Concept - All Ages

```
log.plot.df = log.effects.df$Condition
log.plot <- ggplot(log.plot.df,
                   aes(x = expert_type, y = fit, shape = Condition)) +
  geom_pointrange(aes(ymin = lower, ymax = upper, shape = Condition),
                  position = position_dodge(0.6), size =1) +
  xlab("Abstract Concept") +
  ylab("Proportion Correct Expert Choice") +
  geom_hline(linetype = "dashed", yintercept = 0.5) + ylim(0,1)
log.plot
```

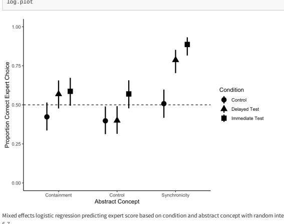

Mixed effects logistic regression predicting expert score based on condition and abstract concept with random intercepts fitted by participant - Age 6-7

```
log.model.younger <- glmer(expert_correct ~ 1 + Condition*expert_type + (1 | Participant),
                   data = df.expert %>% filter(Condition != "Expert Only Control") %>% filter(Age
                   family = binomial(link = "logit"))
log.model.younger %>%
  summary()

# Calculating odds ratios
broom.mixed::tidy(log.model.younger, exponentiate=TRUE, conf.int=TRUE) %>%
  mutate_if(is.numeric, round, 9)

# Predicted effects
log.effects.younger <- allEffects(log.model.younger)
log.effects.younger.df <- lapply(log.effects.younger, as.data.frame)
log.effects.younger.df
```

```
Generalized linear mixed model fit by maximum likelihood (Laplace
  Approximation) [glmerMod]
 Family: binomial  ( logit )
Formula: expert_correct ~ 1 + Condition * expert_type + (1 | Participant)
   Data: df.expert %>% filter(Condition != "Expert Only Control") %>%
    filter(Age < 95)

     AIC      BIC   logLik deviance df.resid
   673.8    716.3   -326.9    653.8      506

Scaled residuals:
    Min      1Q  Median      3Q     Max
-2.5005 -0.9337  0.4101  0.8911  1.5617

Random effects:
 Groups      Name        Variance Std.Dev.
 Participant (Intercept) 0.05375  0.2318
Number of obs: 516, groups:  Participant, 78

Fixed effects:
                                             Estimate Std. Error z value
(Intercept)                                   -0.2060     0.2692  -0.765
ConditionDelayed Test                          0.4861     0.3807   1.277
ConditionImmediate Test                        0.2827     0.3828   0.738
expert_typeControl                             0.3505     0.3755   0.933
expert_typeSynchronicity                       0.4918     0.3769   1.305
ConditionDelayed Test:expert_typeControl      -1.3603     0.5412  -2.514
ConditionImmediate Test:expert_typeControl    -0.6409     0.5357  -1.196
ConditionDelayed Test:expert_typeSynchronicity    0.4834     0.5597   0.864
ConditionImmediate Test:expert_typeSynchronicity  1.2469     0.6015   2.073
                                             Pr(>|z|)
(Intercept)                                    0.4442
ConditionDelayed Test                          0.2016
ConditionImmediate Test                        0.4602
expert_typeControl                             0.3506
expert_typeSynchronicity                       0.1920
ConditionDelayed Test:expert_typeControl       0.0119 *
ConditionImmediate Test:expert_typeControl     0.2315
ConditionDelayed Test:expert_typeSynchronicity    0.3877
ConditionImmediate Test:expert_typeSynchronicity  0.0382 *
---
Signif. codes:  0 '***' 0.001 '**' 0.01 '*' 0.05 '.' 0.1 ' ' 1

Correlation of Fixed Effects:
           (Intr) CndtDT CndtIT expr_C expr_S CDT:_C CIT:_C CDT:_S
CndtnDlydTs -0.704
CndtnImmdtT -0.701  0.494
exprt_typCn -0.699  0.494  0.492
exprt_typSy -0.696  0.493  0.490  0.500
CndtnDTs:_C  0.484 -0.689 -0.343 -0.695 -0.349
CndtnITs:_C  0.489 -0.347 -0.699 -0.701 -0.351  0.489
CndtnDTs:_S  0.469 -0.666 -0.329 -0.336 -0.672  0.467  0.235
CndtnITs:_S  0.437 -0.308 -0.621 -0.312 -0.624  0.214  0.443  0.422
# A tibble: 10 x 9
   effect group term    estimate std.error statistic  p.value conf.low conf.high
   <chr>  <chr> <chr>      <dbl>     <dbl>     <dbl>    <dbl>    <dbl>     <dbl>
 1 fixed  <NA>  (Inter…    0.814     0.219    -0.765   0.444    0.480      1.38
 2 fixed  <NA>  Condit…    1.63      0.619     1.28    0.202    0.771      3.43
 3 fixed  <NA>  Condit…    1.33      0.508     0.738   0.460    0.627      2.81
 4 fixed  <NA>  expert…    1.42      0.533     0.933   0.351    0.680      2.96
 5 fixed  <NA>  expert…    1.64      0.616     1.30    0.192    0.781      3.42
 6 fixed  <NA>  Condit…    0.257     0.139    -2.51    0.0119   0.0888     0.741
 7 fixed  <NA>  Condit…    0.527     0.282    -1.20    0.232    0.184      1.51
 8 fixed  <NA>  Condit…    1.62      0.908     0.864   0.388    0.541      4.86
 9 fixed  <NA>  Condit…    3.48      2.09      2.07    0.0382   1.07      11.3
10 ran_pa… Part… sd__(I…   0.232    NA        NA      NA       NA        NA
$`Condition:expert_type`
       Condition   expert_type       fit         se     lower     upper
1        Control   Containment 0.4486889 0.06659444 0.3240015 0.5797332
2   Delayed Test   Containment 0.5695660 0.06627990 0.4378724 0.6921016
3 Immediate Test   Containment 0.5191717 0.06815097 0.3873759 0.6483524
4        Control       Control 0.5360671 0.06683631 0.4055944 0.6617823
5   Delayed Test       Control 0.3252533 0.06270059 0.2159047 0.4576584
6 Immediate Test       Control 0.4467835 0.06775605 0.3206123 0.5802034
7        Control Synchronicity 0.5709755 0.06634625 0.4390522 0.6935298
8   Delayed Test Synchronicity 0.7782184 0.05532719 0.6518145 0.8680244
9 Immediate Test Synchronicity 0.8600140 0.04684615 0.7413016 0.9294367
```

Expert Detetion Performance by Condition and Abstract Concept - Age 6-7

```
log.plot.younger.df = log.effects.younger.df$Condition
log.plot.younger <- ggplot(log.plot.younger.df,
                   aes(x = expert_type, y = fit, shape = Condition)) +
  geom_pointrange(aes(ymin = lower, ymax = upper, shape = Condition),
                  position = position_dodge(0.6), size =1) +
  xlab("Abstract Concept") +
  ylab("Proportion Correct Expert Choice") +
  geom_hline(linetype = "dashed", yintercept = 0.5) + ylim(0,1)
log.plot.younger
```

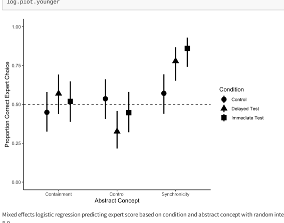

Mixed effects logistic regression predicting expert score based on condition and abstract concept with random intercepts fitted by participant - Age 8-9

```
log.model.older <- glmer(expert_correct ~ 1 + Condition*expert_type + (1 | Participant),
                   data = df.expert %>% filter(Condition != "Expert Only Control") %>% filter(Age
                   family = binomial(link = "logit"))
log.model.older %>%
  summary()

# Calculating odds ratios
broom.mixed::tidy(log.model.older, exponentiate=TRUE, conf.int=TRUE) %>%
  mutate_if(is.numeric, round, 9)

# Predicted effects
log.effects.older <- allEffects(log.model.older)
log.effects.older.df <- lapply(log.effects.older, as.data.frame)
log.effects.older.df
```

```
Generalized linear mixed model fit by maximum likelihood (Laplace
  Approximation) [glmerMod]
 Family: binomial  ( logit )
Formula: expert_correct ~ 1 + Condition * expert_type + (1 | Participant)
   Data: df.expert %>% filter(Condition != "Expert Only Control") %>%
    filter(Age > 95)

     AIC      BIC   logLik deviance df.resid
   674.1    717.0   -327.1    654.1      530

Scaled residuals:
    Min      1Q  Median      3Q     Max
-3.3243 -0.9231  0.3008  0.8094  1.6434

Random effects:
 Groups      Name        Variance Std.Dev.
 Participant (Intercept) 0.0456   0.2136
Number of obs: 540, groups:  Participant, 79

Fixed effects:
                                             Estimate Std. Error z value
(Intercept)                                   -0.4102     0.2681  -1.530
ConditionDelayed Test                          0.6159     0.3763   1.636
ConditionImmediate Test                        0.8944     0.3806   2.350
expert_typeControl                            -0.5279     0.3914  -1.349
expert_typeSynchronicity                       0.2750     0.3715   0.740
ConditionDelayed Test:expert_typeControl       0.2575     0.5373   0.479
ConditionImmediate Test:expert_typeControl     0.7480     0.5481   1.365
ConditionDelayed Test:expert_typeSynchronicity    0.8201     0.5526   1.484
ConditionImmediate Test:expert_typeSynchronicity  1.6612     0.6548   2.537
                                             Pr(>|z|)
(Intercept)                                    0.1260
ConditionDelayed Test                          0.1017
ConditionImmediate Test                        0.0188 *
expert_typeControl                             0.1775
expert_typeSynchronicity                       0.4591
ConditionDelayed Test:expert_typeControl       0.6318
ConditionImmediate Test:expert_typeControl     0.1724
ConditionDelayed Test:expert_typeSynchronicity    0.1378
ConditionImmediate Test:expert_typeSynchronicity  0.0112 *
---
Signif. codes:  0 '***' 0.001 '**' 0.01 '*' 0.05 '.' 0.1 ' ' 1

Correlation of Fixed Effects:
           (Intr) CndtDT CndtIT expr_C expr_S CDT:_C CIT:_C CDT:_S
CndtnDlydTs -0.712
CndtnImmdtT -0.703  0.503
exprt_typCn -0.667  0.474  0.468
exprt_typSy -0.706  0.504  0.498  0.482
CndtnDTs:_C  0.487 -0.683 -0.342 -0.728 -0.352
CndtnITs:_C  0.476 -0.338 -0.675 -0.715 -0.344  0.520
CndtnDTs:_S  0.472 -0.663 -0.331 -0.326 -0.671  0.466  0.233
CndtnITs:_S  0.398 -0.282 -0.564 -0.275 -0.566  0.200  0.396  0.383
# A tibble: 10 x 9
   effect group term    estimate std.error statistic  p.value conf.low conf.high
   <chr>  <chr> <chr>      <dbl>     <dbl>     <dbl>    <dbl>    <dbl>     <dbl>
 1 fixed  <NA>  (Inter…    0.664     0.178    -1.53    0.126    0.392      1.12
 2 fixed  <NA>  Condit…    1.85      0.697     1.64    0.102    0.885      3.87
 3 fixed  <NA>  Condit…    2.45      0.931     2.35    0.0188   1.16       5.16
 4 fixed  <NA>  expert…    0.590     0.231    -1.35    0.177    0.274      1.27
 5 fixed  <NA>  expert…    1.32      0.489     0.740   0.459    0.636      2.73
 6 fixed  <NA>  Condit…    1.29      0.695     0.479   0.632    0.451      3.71
 7 fixed  <NA>  Condit…    2.11      1.16      1.36    0.172    0.722      6.19
 8 fixed  <NA>  Condit…    2.27      1.25      1.48    0.138    0.769      6.71
 9 fixed  <NA>  Condit…    5.27      3.45      2.54    0.0112   1.46      19.0
10 ran_pa… Part… sd__(I…   0.214    NA        NA      NA       NA        NA
$`Condition:expert_type`
       Condition   expert_type       fit         se     lower     upper
1        Control   Containment 0.3988696 0.06427881 0.2817882 0.5287818
2   Delayed Test   Containment 0.5512465 0.06533894 0.4226321 0.6733541
3 Immediate Test   Containment 0.6187499 0.06384916 0.4884407 0.7339461
4        Control       Control 0.2812902 0.05894966 0.1810015 0.4093715
5   Delayed Test       Control 0.4838261 0.06561816 0.3590030 0.6107005
6 Immediate Test       Control 0.6691422 0.06184934 0.5391096 0.7776212
7        Control Synchronicity 0.4662579 0.06549096 0.3427727 0.5940214
8   Delayed Test Synchronicity 0.7859700 0.05372078 0.6625985 0.8728847
9 Immediate Test Synchronicity 0.9183726 0.03544827 0.8166230 0.9660144
```

Expert Detetion Performance by Condition and Abstract Concept - Age 8-9

```
log.plot.older.df = log.effects.older.df$Condition
log.plot.older <- ggplot(log.plot.older.df,
                   aes(x = expert_type, y = fit, shape = Condition)) +
  geom_pointrange(aes(ymin = lower, ymax = upper, shape = Condition),
                  position = position_dodge(0.6), size =1) +
  xlab("Abstract Concept") +
  ylab("Proportion Correct Expert Choice") +
  geom_hline(linetype = "dashed", yintercept = 0.5) + ylim(0,1)
log.plot.older
```

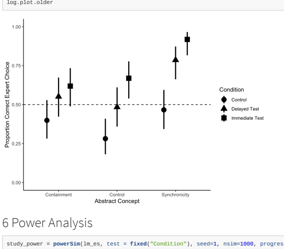

6 Power Analysis

```
study_power = powerSim(lm_es, test = fixed("Condition"), seed=1, nsim=1000, progress=FALSE)
```

```
Warning in observedPowerWarning(sim): This appears to be an "observed power"
calculation
```

```
print(study_power)
```

```
Power for predictor 'Condition', (95% confidence interval):
      93.70% (92.01, 95.13)

Test: Likelihood ratio

Based on 1000 simulations, (1000 warnings, 0 errors)
alpha = 0.05, nrow = 180

Time elapsed: 0 h 0 m 4 s

nb: result might be an observed power calculation
```

7 Adult Study

Adult Study Summary: Mean age of understanding by item

```
#read in data file
df.adult = read_csv("data/ENG_adultstudy_data.csv")

#factor Condition levels
df.byitem = df.adult %>%
  pivot_longer(cols = "How a computer works":"How a car engine works", names_to = "item", values_to = "age.
  group_by(item) %>%
  summarize(age_of_understanding = mean(age.of.understanding)) %>%
  transform(item=reorder(item, -age_of_understanding))

df.byitem %>%
  kable()
```

item	age_of_understanding
A soccer game	7.000000
Coral reefs	9.268293
Emperor penguins	7.292683
Fidget spinners	5.634146
Game of thrones	12.219512
How a bill becomes a law	9.926829
How a car engine works	12.317073
How a computer works	10.804878
How bread is made	8.073171
How dinosaurs went extinct	8.048780
How paper is made	8.219512
How to ride a bike	5.609756
Pokemon	5.975610
Snowstorms	6.341463
Spongebob	4.609756
Teletubbies	3.756098
The American Flag	7.317073
The history of Spain	10.926829
The industrial revolution	10.902439
The periodic table	11.463415
The video game, Fortnite	7.634146
Where money comes from	9.926829

Adult study results by item

```
ggplot(data=df.byitem,
       aes(x = item,
           y = age_of_understanding)
       ) +
  geom_bar(position="dodge", stat="identity") +
  theme_classic() +
  scale_fill_brewer(palette="Greys") +
  labs(y = "At what age would a majority of children understand?",
       x = "A video about/an episode of...") +
  coord_flip()
```

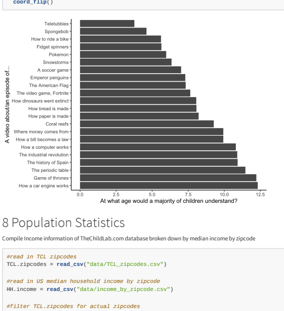

8 Population Statistics

Compile Income information of TheChildLab.com database broken down by median income by zipcode

```
#read in TCL zipcodes
TCL.zipcodes = read_csv("data/TCL_zipcodes.csv")

#read in US median household income by zipcode
HH.income = read_csv("data/income_by_zipcode.csv")

#filter TCL.zipcodes for actual zipcodes
TCL.zipcodes = TCL.zipcodes %>%
  filter(zipcode %in% HH.income$Zip.Code)

#reformat HH.income
HH.income = HH.income %>%
  pivot_wider(names_from = Zip.Code, values_from = Median.Income)

#create lookup function
lookup <- function(x) {
  return(HH.income[[x]])
}

#look up TCL zipcodes' median incomes
TCL.zipcodes = TCL.zipcodes %>%
  mutate(median_income = lapply(zipcode, lookup) %>%
           unlist() %>%
           as.numeric()
         )
```

Produce general income information

```
TCL.zipcodes %>%
  summarise(mean_income = mean(median_income),
            median_median_income = median(median_income),
            sd_income = sd(median_income),
            lowest_income = min(median_income),
            highest_income = max(median_income),
            total_participants = length(zipcode),
            unique_zipcodes = length(unique(zipcode))
            )
```

```
# A tibble: 1 x 7
  mean_income median_median_i… sd_income lowest_income highest_income
        <dbl>            <dbl>     <dbl>         <dbl>          <dbl>
1      72545.            65821    28027.         13468         200001
# … with 2 more variables: total_participants <int>, unique_zipcodes <int>
```

Generage density plot of median incomes

```
ggplot(TCL.zipcodes, aes(x = median_income)) +
  geom_histogram(aes(y=..density..), binwidth = 3000) +
  geom_density(alpha = .5, size = 2, color = "red")
```

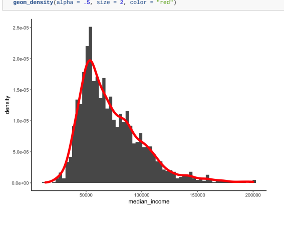

Supplement: Supplementary file 3 — Supplementary Information [file 41539_2021_108_MOESM3_ESM.pdf]
